# High Prevalence of Laxative Use Among Those Tested for Clostridioides difficile Infection in VA Hospitals

**DOI:** 10.1017/ash.2024.211

**Published:** 2024-09-16

**Authors:** Geneva Wilson, Lishan Cao, Margaret Fitzpatrick, Katie Suda, Charlesnika Evans

**Affiliations:** Edward Hines Jr. VA Hospital; None; University of Colorado Anschutz Medical Center; University of Pittsburgh School of Medicine; Northwestern University, Feinberg School of Medicine

## Abstract

**Background:** Clostridioides difficile infection (CDI) is associated with 500,000 infections and 30,000 deaths per year. Inappropriate testing and treatment of patients with asymptomatic colonization occurs frequently (between 15% and 41%). The VA CDI guidelines emphasize avoidance of CDI testing in patients with laxative use within the previous 48 hours due to the high likelihood of non-infectious diarrhea. The objective of this study was to assess laxative administration among inpatients tested for CDI in VA hospitals and identify factors associated with guideline discordance. **Methods:** Adults hospitalized in Illinois, Wisconsin, and Michigan VA Medical Centers from January 2019-December 2022 with a CDI test performed during the admission were included. CDI tests included Toxin B gene Polymerase Chain Reaction or Toxin Enzyme Immunoassay. Tests were defined as positive, negative, or cancelled according to the diagnostic protocols of the VA testing laboratories. Laxative use, patient demographics, admission data, and comorbidities were collected from the VA Corporate Data Warehouse. Guideline discordant testing was defined as a diagnostic test for CDI ordered within 48 hours of a recorded laxative dose. Factors associated with discordant testing were analyzed using clustered binomial logistic regression models. Analyses were completed using SAS 9.4. **Results:** There were 7,326 tests ordered for 4,888 patients during the study. Patients were predominantly White (61.8%), male (95.6%), and elderly (mean age=70.0 standard deviation=12.1). Most (59.0%) patients had received at least one dose of laxative in the 48 hours preceding their CDI test. Being Black (Odds Ratio (OR)=0.86 (95%Confidence Interval (95%CI) =0.76,0.98) or Hispanic (OR (95%CI) =0.62(0.48,0.82) vs White) was associated with a decreased likelihood of inappropriate testing due to recent laxative use. Being tested at a rural facility (OR (95%CI) =1.23 (1.07,1.41) vs urban), within a long-term care (LTC) unit (OR (95%CI) =1.67 (1.41,1.97) vs inpatient), or within an intensive care unit (ICU) (OR (95%CI) =1.40 (1.24,1.59)) were all associated with an increased likelihood of being inappropriately tested. Guideline discordant tests were more likely to have negative results (OR (95%CI) =1.25 (1.05,1.49)) compared to guideline concordant tests. Discussion: Laxative administration in the 48 hours preceding CDI testing was common among hospitalized Veterans and associated with a lower likelihood of positive **Results:** This echoes non-VA studies where laxative use was reported at 44%. An increased likelihood of guideline discordant testing in ICU and LTC settings suggests the need for greater diagnostic stewardship interventions. Additionally, further work to determine negative outcomes associated with inappropriate testing are needed.

